# Adjustable Autologous Pubovaginal Sling for Complex Stress Urinary Incontinence: A Technical Surgical Video with a Case Series

**DOI:** 10.1007/s00192-026-06595-7

**Published:** 2026-06-14

**Authors:** Marisa N. Duong, Christina M. Mezes, Amr S. El Haraki, Catherine A. Matthews

**Affiliations:** 1 Department of Obstetrics and Gynecology, Wake Forest University School of Medicine, 1 Medical Center Boulevard, Winston Salem, NC 27157 USA; 2 Division of Urogynecology, Department of Urology, Wake Forest University School of Medicine, Winston Salem, NC USA

**Keywords:** Adjustable sling, Autologous sling, Stress urinary incontinence

## Abstract

**Introduction and Hypothesis:**

Autologous pubovaginal slings are an important option for the management of stress urinary incontinence (SUI) but are associated with higher risks of voiding dysfunction. There is no standardized method for tensioning that balances the effectiveness of treatment with the prevention of voiding dysfunction.

**Methods:**

We present a video demonstrating the surgical steps of a novel combination of a fascia lata sling combined with an adjustable tensioning system, as well as an accompanying case series of short-term outcomes. The video demonstrates the use of this system for a 59-year-old female with chronic pain and tobacco use who presented with severe, recurrent SUI after Burch colposuspension.

**Results:**

In 2025, a total of 9 cases of fascial slings combined with the adjustable tensioning system were performed at this institution, with all patients demonstrating a negative cough stress test after tensioning and with no cases of postoperative urinary retention or voiding dysfunction at least 6 months postoperatively. Data collection for long-term outcomes is ongoing.

**Conclusion:**

The combination of an autologous fascial sling with a personalized adjustable tensioning system offers a unique modification to a commercially available system that may provide women with complex SUI and risk factors for mesh complications with a feasible way of achieving continence while mitigating the risks of voiding dysfunction typically seen with a fascial sling.

**Supplementary Information:**

The online version contains supplementary material available at https://doi.org/10.1007/s00192-026-06595-7

## Introduction

Autologous pubovaginal slings are efficacious for the management of stress urinary incontinence (SUI), with success rates of 66% compared with 49% with Burch colposuspension [1]. However, they are associated with higher rates of urinary tract infections, difficulty voiding, and postoperative urge incontinence [1]. Despite these risks, they serve an important role in cases of recurrent SUI or when synthetic mesh is contraindicated.

Various techniques are used for tensioning fascial slings, including visual estimation, Crede maneuvers, or use of a spacer [2]. However, there is no consensus on which yields the best outcomes, and up to one third of fascial slings are overtight [1]. The adjustable midurethral sling (Remeex; Neomedic International, Terrassa, Spain) utilizes a permanent subcutaneous suprafascial “varitensor,” allowing tensioning immediately postoperatively and in the long term. Studies cite effectiveness rates of up to 70–90% [3–8].

In cases of recurrent SUI, in which nuanced tensioning may be required to identify the ideal balance between persistent SUI versus voiding dysfunction, we hypothesize that combining a fascial sling with this tensioning system may provide better outcomes. The purpose of this video is to present our surgical technique in an individual case and provide results regarding our short-term outcomes from a case series.

## Case Presentation

A 59-year-old female with chronic pain, prior spinal surgeries, and heavy tobacco use presented with recurrent SUI. She had undergone hysterectomy, prolapse repair, and Burch colposuspension 25 years prior. She demonstrated a large-volume positive empty supine cough stress test and urethral hypermobility without prolapse. Given the mesh complication risk and symptom severity, the decision was made to perform a pubovaginal autologous fascial sling procedure with the adjustable system for personalized tensioning. This study was deemed exempt by the institutional review board, IRB00128886, as a video case report and retrospective review.

## Surgical Technique

The patient was positioned in dorsal lithotomy with internal rotation of the left leg to facilitate fascia lata harvesting. Bony landmarks of the upper leg were marked, including the lateral epicondyle and greater trochanter. A 4-cm incision was made 5 cm superior to the lateral femoral epicondyle and taken down to the fascia using electrocautery. An incision was made at the inferior border of the fascia, extended superiorly, and transected to produce a fascial strip measuring 2.5 by 10 cm. We have since modified our technique to utilize a shorter strip (2.5 by 6 cm) to minimize scarring in the retropubic space and facilitate future tensioning. The ends of the fascia were tagged with 0 polypropylene suture, and the midportion with polydioxanone suture. The fascial strip was then soaked in saline.

The left leg was externally rotated, and a lone star retractor was placed with the smaller ring over the vagina. Local anesthetic was injected into the anterior vaginal wall at the level of the midurethra followed by an inverted “U” incision. The vaginal epithelium was dissected from the pubocervical fascia to the bladder neck. Paraurethral dissection was performed through the perineal membrane towards the pubic rami. The bladder was swept from the posterior pubic symphysis, which is often required owing to scar tissue from prior retropubic procedures. The perineal membrane was opened to accommodate the surgeon’s finger.

Turning our attention to the abdomen, a 3-cm incision was made 2 cm cephalad to the pubic bone, and dissection to the rectus fascia was performed using electrocautery. With a finger in the paraurethral dissection, a Stamey needle was passed from the abdominal incision to the receiving finger in the retropubic dissection. Care was taken to ensure that the needle did not drift laterally to avoid the external iliac vessels. This was repeated on the contralateral side. The adjustable sling includes trocars, but we prefer the use of Stamey needles because of their smaller diameter.

Cystoscopy confirmed no trauma or perforation of the bladder or urethra, and the Foley catheter was replaced. The sutures attached to the ends of the fascial sling were passed through the Stamey needles and brought up through the abdominal incision. The fascial sling was anchored to the mid-urethra using delayed absorbable suture at 6 and 12 o’clock. The sutures were passed through the “varitensor,” and the manipulator was used to tighten the sutures, leaving two fingerbreadths between the device and the fascia. The device was tucked into the incision with the manipulator left externally for later adjustment.

The suprapubic incision was closed with subcutaneous sutures followed by 2–0 polyamide interrupted sutures around the device. The incision was covered with open gauze sponges and tape. The vaginal incision was closed with 2–0 polyglactin sutures, and the leg incision was closed with subcutaneous absorbable sutures followed by 2–0 polyamide horizontal mattress skin sutures. The leg incision was covered with a non-adherent bandage and compression wrap for 72 h.

## Results

The patient returned to the clinic on postoperative day 1 for sling tensioning. After backfilling the bladder, she performed a standing cough stress test. The device was tightened until no further leakage was noted. A standardized office void trial was performed by backfilling the bladder with 300 ml of normal saline. After confirming the absence of voiding dysfunction defined as a postvoid residual (PVR) volume less than 150 ml, the external manipulator was removed.

Her postoperative course was complicated by muscle herniation at the site of the fascia lata harvest that resolved with manual reduction, ice, and use of a compression bandage. At 7 weeks, she reported normal voiding without incontinence. At 6 months, she had no SUI but reported urinary hesitancy with a PVR of 0 cc. The decision was made not to adjust the sling further at that time. However, future adjustments remain feasible owing to the ease of accessing the suprapubic implant in the operating room.

## Case Series

To date, a total of 9 women with complex SUI defined as recurrent SUI or intrinsic sphincter deficiency have undergone the modified adjustable fascial sling procedure. This specific modified procedure was not offered to any patients with neurogenic bladder, detrusor underactivity, or voiding dysfunction defined as an elevated PVR (> 150 cc). Two of the nine reported urge incontinence at their initial consultations, with urodynamic testing confirming reduced bladder capacity and overactive detrusor activity.

There were no intraoperative complications including hemorrhage, bladder, or urethral injury. Postoperatively, there was one case of muscle herniation at the site of fascia lata harvest, as described above. There was one case of uncomplicated postoperative urinary tract infection and one case of a superficial skin infection at the suprapubic incision site, which resolved with oral antibiotics. The superficial surgical site infection was specifically in a patient with risk factors, including type 2 diabetes mellitus and morbid obesity. Six out of nine required tightening of the sling using the varitensor device on post-operative day 1 to achieve a negative standing cough stress test after backfilling the bladder with 300 ml. One hundred percent were able to void to completion (PVR < 50 ml) following tensioning. Subjective urinary incontinence was assessed at each postoperative visit as a standardized question, with all patients reporting no stress incontinence. The two patients with mixed urinary incontinence did report ongoing mild urge incontinence postoperatively. There have been no re-operations to date. Data collection for long-term outcomes is still ongoing. However, in another patient with recurrent SUI who had the same device placed 10 years prior at another institution utilizing synthetic mesh under the urethra, we re-accessed the original device and tightened the sling to resolve her symptoms.

Another case in the literature described placement of an adjustable fascial sling using rectus fascia in a patient with prior mesh sling complications. This case demonstrated immediate resolution of incontinence, although long-term outcomes were not described [9]. In our video, we used fascia lata to mitigate risks of seroma or hematoma formation at the implant site. Although rectus fascia is more commonly used, it has been associated with incisional hernias and seromas [10]. Although fascia lata harvest can lead to postoperative leg discomfort or muscle herniation, as in this patient, the majority of patients report that symptoms are not bothersome [11]. Prior studies using the adjustable sling system with the included mesh sling have reported rates of wound infection of 1.5–7.4%, with at least four cases requiring removal of the suprapubic implant specifically because of infection. Given the permanent suprapubic device placement, avoiding wound complications is paramount, and the use of fascia lata instead of rectus fascia may help to mitigate these risks [3, 4, 6, 8, 12].

## Conclusion

This video demonstrates the use of an autologous pubovaginal sling with an adjustable device for personalized tensioning. This modification may mitigate the risk of voiding dysfunction typically seen with fascial slings. Results of this technique thus far have been promising, and this method of tensioning a fascial sling may provide those with severe SUI or contraindications to mesh a feasible way of achieving their goal of being dry without compromising their ability to void.

## Supplementary Information

Below is the link to the electronic supplementary material.

Supplementary file1 (MP4 98905 KB)
